# Chloroquine has tumor-inhibitory and tumor-promoting effects in triple-negative breast cancer

**DOI:** 10.3892/ol.2013.1602

**Published:** 2013-10-04

**Authors:** JOHANNA TUOMELA, JOUKO SANDHOLM, JOONAS H. KAUPPILA, PETRI LEHENKARI, KEVIN W. HARRIS, KATRI S. SELANDER

**Affiliations:** 1 Department of Medicine, University of Alabama at Birmingham, Birmingham, AL 35294, USA; 2 MediCity/PET Preclinical Imaging, Turku PET Centre, University of Turku, Turku 20521, Finland; 3 Turku Centre for Biotechnology, University of Turku and Abo Akademi University, Turku 20520, Finland; 4 Department of Anatomy and Cell Biology, University of Oulu, Oulu 90014, Finland; 5 Department of Surgery, University Hospital of Oulu, Oulu 90020, Finland; 6 Department of Pathology, University Hospital of Oulu, Oulu 90020, Finland; 7 Birmingham Veterans Affairs Medical Center, Birmingham, AL 35233, USA

**Keywords:** chloroquine, hypoxia, toll-like receptor-9, triple-negative breast cancer

## Abstract

Toll-like receptor-9 (TLR9) is an intracellular DNA receptor that is widely expressed in breast and other cancers. We previously demonstrated that low tumor TLR9 expression upon diagnosis is associated with significantly shortened disease-specific survival times in patients with triple-negative breast cancer (TNBC). There are no targeted therapies for this subgroup of patients whose prognosis is among the worst in breast cancer. Due to the previously detected *in vitro* anti-invasive effects of chloroquine in these cell lines, the present study aimed to investigate the *in vivo* effects of chloroquine against two clinical subtypes of TNBC that differ in TLR9 expression. Chloroquine suppressed matrix metalloproteinase (MMP)-2 and MMP-9 mRNA expression and protein activity, whereas MMP-13 mRNA expression and proteolytic activity were increased. Despite enhancing TLR9 mRNA expression, chloroquine suppressed TLR9 protein expression *in vitro*. Daily treatment of mice with intraperitoneal (i.p.) chloroquine (80 mg/kg/day) for 22 days, did not inhibit the growth of control siRNA or TLR9 siRNA MDA-MB-231 breast cancer cells. In conclusion, despite the favorable *in vitro* effects on TNBC invasion and viability, particularly in hypoxic conditions, chloroquine does not prevent the growth of the triple-negative MDA-MB-231 cells with high or low TLR9 expression levels *in vivo*. This may be explained by the activating effects of chloroquine on MMP-13 expression or by the fact that chloroquine, by suppressing TLR9 expression, permits the activation of currently unknown molecular pathways, which allow the aggressive behavior of TNBC cells with low TLR9 expression in hypoxia.

## Introduction

Of all breast cancer patients, those with triple-negative tumors that lack the expression of the estrogen receptor (ER), progesterone receptor and Her2 receptor, bear the poorest prognoses (1). This is due to the aggressive behavior of triple-negative breast cancer (TNBC) cells and the lack of targeted therapies for this particular subgroup. TNBC is, however, a highly heterogeneous disease and more specific information concerning the biology of the various subtypes is required for future targeted therapies (1,2).

Toll-like receptor-9 (TLR9) is an innate immunity DNA receptor that was first identified in cells of the immune system (3). Agonistic TLR9 ligands, such as microbial and vertebrate DNA or synthetic CpG-sequence-containing oligonucleotides, induce an inflammatory reaction in cells that express TLR9. In addition to inducing the release of cytokines (4,5) in cancer cells, TLR9 agonists also induce invasion *in vitro*, which is mediated via the activation of matrix metalloproteinases (MMPs), such as MMP-13 (6–8). We previously demonstrated that TLR9 has an important role in TNBC (9) and showed that, while low tumor TLR9 expression was associated with significantly shortened breast cancer-specific survival in patients with TNBC, TLR9 had no prognostic value in breast cancer patients with ER^+^ tumors (9). This is likely to be, at least partly, explained by the hypoxia-associated behavior of TNBC cells that express low TLR9 levels. We revealed that, although decreased TLR9 expression in TNBC cells results in decreased invasion when the tumor cells are in normoxia, the cells become highly invasive in hypoxia (9). These results suggested that TLR9 also has ligand-independent effects on invasion and that in the absence of TLR9 expression in hypoxia, another pathway is the actual mediator of invasion. The pathway that mediates invasion in hypoxia in the absence of TLR9 is not currently known. It is also not known whether possible impaired TLR9-mediated inflammation at the site of the tumor contributes to the poor prognosis in this subgroup of TNBC.

Since the hypoxia-induced *in vitro* invasion and viability of TNBC cells expressing low levels of TLR9 was inhibited *in vitro* by chloroquine (9), a well-established malaria and rheumatoid arthritis drug that is known to interfere with endosomal signaling, the present study aimed to further characterize the anti-tumor efficacy of chloroquine against TNBC cells with differences in TLR9 expression.

## Materials and methods

### Cell culture

Parental MDA-MB-231 breast cancer cells and D54MG, U373MG, Caco-2 and AGS cells were cultured in Dulbecco’s modified Eagle’s medium (Gibco BRL, Life Technologies, Carlsbad, CA, USA) supplemented with 10% heat-inactivated fetal bovine serum, L-glutamine, penicillin/streptomycin and non-essential amino acids (all from Gibco BRL, Life Technologies) (10). The cells were cultured in incubators at 37°C with an atmosphere of 5% CO_2_/95% air with ~21% pO_2_ or in a hypoxia incubator with 5% pO_2_ (I-Glove; BioSpherix, Ltd., Lacona, NY, USA). The stable control siRNA and TLR9 siRNA MDA-MB-231 cells have been described previously and were cultured in the presence of G418 (800 μg/ml) (9). Chloroquine was purchased from Sigma (St. Louis, MO, USA).

### RNA isolation and quantitative (q)PCR

Total RNA was isolated from the cells using the TRIzol reagent (Invitrogen Life Technologies, Carlsbad, CA, USA) and purified with RNeasy mini kits (Qiagen, Hilden, Germany). All reagents for the qPCR experiments were purchased from Applied Biosystems (Foster City, CA, USA). cDNA was synthesized from 0.2 μg total RNA, using Multiscribe Reverse Transcriptase and random hexamers. Quantification of TLR9 mRNA expression was performed as previously described (11). The other primer and probe sets that were used (MMP-2, MMP-9, MMP-13 and TIMP-3) were purchased from Applied Biosystems as ready-made primer/probe sets. A standard amplification program was used for all amplifications (1 cycle of 50°C for 2 min, 1 cycle of 95°C for 10 min, 40 cycles of 95°C for 15 sec and 60°C for 1 min). Subsequent to normalization with ribosomal protein L15 (RPLO) expression levels for each cDNA, relative quantification of target cDNA was performed using 2^−ΔΔct^ values.

### Western blot analysis

The cells were cultured in 6-well plates with normal culture medium until near confluency, after which they were rinsed with sterile phosphate-buffered saline (PBS) and cultured further for the indicated times in serum-free culture medium. At the desired time-points, the culture medium was discarded and the cells were quickly harvested in lysis buffer (Cell Signaling Technology, Inc., Danvers, MA, USA) and clarified by centrifugation, as previously described (8). Subsequent to boiling the supernatants in reducing sodium dodecyl sulphate (SDS) sample buffer, equal amounts of protein (~100 μg) were loaded per lane and the samples were electrophoresed into 10 or 4–20% gradient polyacrylamide SDS gels (Bio-Rad Laboratories, Inc., Hercules, CA, USA), then transferred to a nitrocellulose membrane. To detect TLR9, the blots were incubated overnight at 4°C with anti-TLR9 antibodies (IMG-431; Imgenex, San Diego, CA, USA), diluted 1:500 in Tris-buffered saline with 0.1% (v/v) Tween-20 (TBST). Equal loading was confirmed with polyclonal rabbit anti-actin (Sigma; A-2066, used at 1:1,000 dilution). Secondary detection was performed with horseradish peroxidase-linked secondary antibodies (GE Healthcare, Piscataway, NJ, USA). The protein bands were visualized by chemiluminescence using an ECL kit (Pierce Biotechnology, Inc., Rockford, IL, USA).

### Cell viability assays

The cells were plated into 96-well plates (20,000 cells per 100 μl per well) in normal growth medium. The viability of the cells was measured with the CellTiter 96 Aqueous One Solution Cell Proliferation assay (Promega Corporation, Madison, WI, USA), according to the manufacturer’s recommendations. In another set of experiments, the cells were plated into 24-well plates and after the indicated time, the cells were trypsinized and the viable cells were counted following trypan blue staining using a TC10™ automated cell counter (Bio-Rad Laboratories).

### Zymography

The cells were incubated for 24–48 h in serum-free media. The supernatants were collected and concentrated using a centrifugal filter device (Millipore, Billerica, MA, USA; cut-off size 3 kDa, cat no. UFC5-003-24). Equal amounts of protein (~20 μg) were loaded per lane of zymogram gels (10% gelatin, Bio-Rad Laboratories). The gels were then run, renaturated, developed and stained using Bio-Rad zymogram buffers, according to the manufacturer’s recommendations.

### Animal studies

Control and TLR9 siRNA MDA-MB-231 cells (5×10^5^ cells in 100 μl) were inoculated into the mammary fat pads of four-week-old, immune-deficient mice (athymic nude/nu Foxn1; Harlan Sprague Dawley, Inc., Indianapolis, IN, USA). Treatments were started seven days after tumor cell inoculation. The mice were treated daily either with intraperitoneal (i.p.) chloroquine (80 mg/kg) or vehicle (PBS). The animals were monitored daily for clinical signs. Tumor measurements were performed twice a week and tumor volume was calculated according to the formula V = (π / 6) (d_1_ × d_2_)^3/2^, where d_1_ and d_2_ are perpendicular tumor diameters (9). The tumors were allowed to grow for 22 days, at which point the mice were sacrificed and the tumors were dissected for a final measurement. Throughout the experiments, the animals were maintained under controlled pathogen-free environmental conditions (20–21ºC, 30–60% relative humidity and a 12-h lighting cycle). The mice were fed with small-animal food pellets (Harlan Sprague Dawley) and supplied with sterile water *ad libitum*. The experimental procedures were reviewed and approved by the University of Alabama at Birmingham Institutional Animal Care and Use Committee.

### Statistical analysis

The results are presented as the mean ± SD or mean ± SEM, as stated. Unpaired Student’s t-tests were used to calculate statistically significant differences between the various study groups in the *in vitro* and pre-clinical *in vivo* experiments.

## Results

### Effects of chloroquine on cellular viability of parental MDA-MB-231 cells

**Figure 1:**
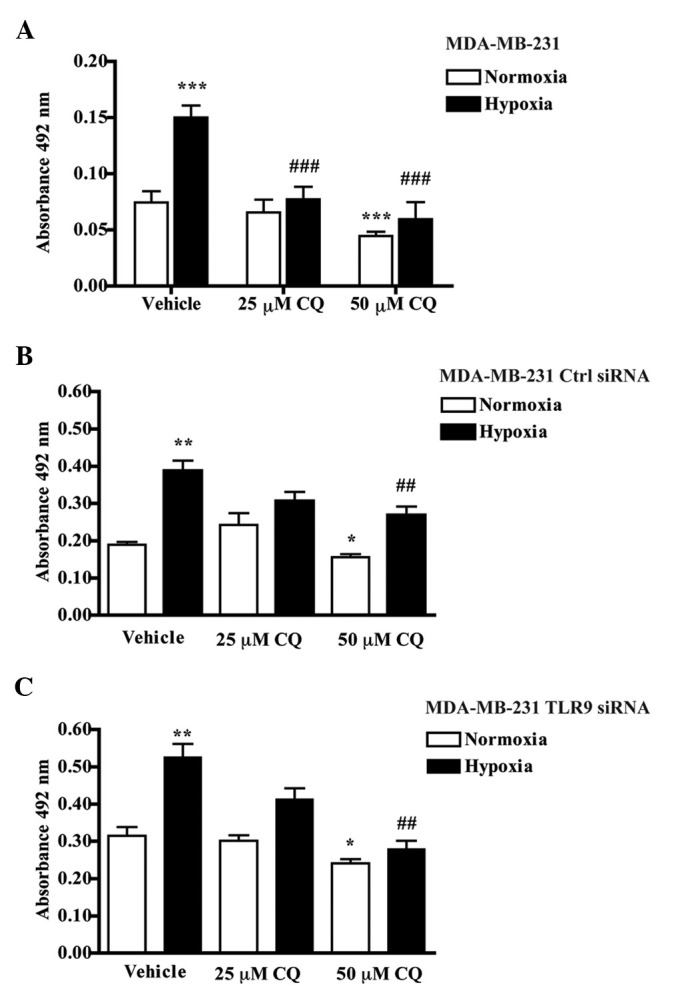
(A) Parental MDA-MB-231, (B) MDA-MB-231 control siRNA and (C) MDA-MB-231 TLR9 siRNA cells were cultured in normoxic (pO_2_ 21%) and hypoxic (pO_2_ 5%) culture conditions for 24 h, after which cellular viability was measured with MTS-assays. Data is expressed as the mean ± SEM, n=6. ^*^P<0.05, ^**^P<0.01 and ^***^P<0.001 vs. vehicle in normoxia; ^##^P<0.01 and ^###^P<0.001 vs. vehicle in hypoxia. TLR9, toll-like receptor-9; MTS, 3-(4,5-dimethylthiazol-2-yl)-5-(3-carboxymethoxyphenyl)-2-(4sulfophenyl)-2H-tetrazolium, inner salt

Since the behavior of TNBC cells is significantly affected by hypoxia (9,12), all experiments were conducted in normoxic (pO_2_ 21%) and hypoxic (pO_2_ 5%) culture conditions. First, the effects of chloroquine on the cellular viability of the parental MDA-MB-231 cells were investigated. In agreement with our previous observations (9), hypoxic culture conditions induced a significant increase in parental MDA-MB-231 cell viability compared with cultures that were kept in normoxia (9). The addition of 25 μM chloroquine did not affect MDA-MB-231 viability in normoxia, whereas 50 μM chloroquine had a slight but significant inhibitory effect. Neither dose of chloroquine, however, completely blocked the hypoxia-induced increase in viability (Fig. 1A). Similar studies were also conducted with MDA-MB-231 cells that were stably transfected with control siRNA- or TLR9 siRNA-encoding plasmids. Chloroquine also inhibited the hypoxia-induced increase in viability in these two cell lines (Fig. 1B and C). Taken together, these results suggest that chloroquine dose-dependently inhibits the hypoxia-induced viability of MDA-MB-231 cells and that these effects are independent of the TLR9 expression status of the cells.

### Effects of chloroquine on hypoxia-induced TLR9 expression

**Figure 2:**
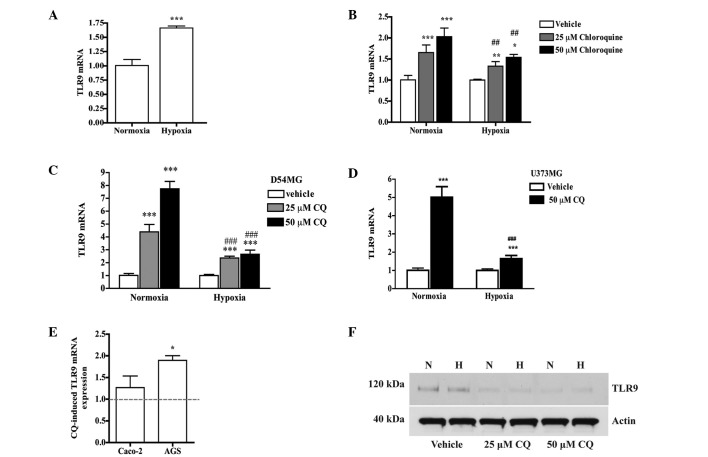
(A) Parental MDA-MB-231 cells were cultured for 24 h under hypoxia and normoxia. Expression of TLR9 mRNA was measured with qPCR; mean ± SEM, n=6. ^***^P<0.001 vs. normoxia. (B) Expression of TLR9 mRNA in parental MDA-MB-231 cells. Bars represent chloroquine-induced changes in TLR9 mRNA expression relative to vehicle treatment in normoxia and hypoxia; mean ± SD, n=6. ^*^P<0.05, ^**^P<0.01 and ^***^P<0.001 vs. the corresponding vehicle; ^##^P<0.01 vs. corresponding chloroquine concentration in normoxia. (C) D54MG and (D) U373MG cells were cultured in normoxia and hypoxia in the presence of vehicle or 25 or 50 μM chloroquine for 24 h, and TLR9 mRNA was measured with qPCR. Data are expressed as fold-change in TLR9 mRNA expression vs. corresponding vehicle. Mean ± SEM, n=6. ^***^P<0.001 vs. vehicle and ^###^P<0.001 vs. corresponding chloroquine-treatment in normoxia. (E) Caco-2 and AGS cells were cultured with 50 μM chloroquine in normoxia; mean ± SEM, n=4. ^*^P<0.05 vs. vehicle (vehicle is set to 1 and represented by the dotted line). (F) Western blot analysis of TLR9 protein in parental MDA-MB-231 cells after culture for 24 h in normoxia (N) and hypoxia (H), in the presence of vehicle or 25 or 50 μM chloroquine. Actin band of the same stripped blot is shown to indicate equal loading. Mean ± SEM, n=6. qPCR, quantitative PCR; CQ, chloroquine; TLR9, toll-like receptor-9.

**Figure 3:**
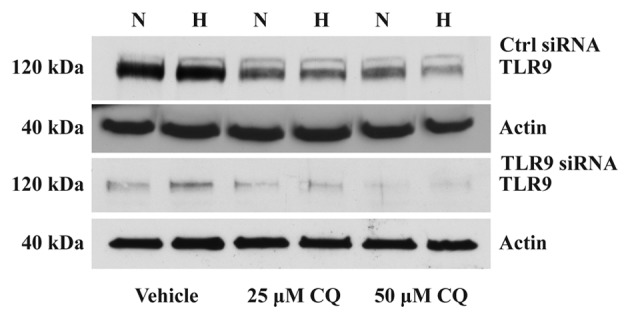
Western blot analysis of TLR9 protein in control siRNA or TLR9 siRNA MDA-MB-231 cells after culture for 24 h under normoxia (N) or hypoxia (H), in the presence of vehicle or 25 or 50 μM chloroquine. Actin band of the same stripped blot is shown to indicate equal loading. TLR9, toll-like receptor-9.

Next, the effects of chloroquine on hypoxia-induced TLR9 expression were studied. As also previously detected, hypoxia induced a significant increase in TLR9 mRNA expression in the parental MDA-MB-231 cells (Fig. 2A). This effect was significantly enhanced by chloroquine in the normoxic and hypoxic culture conditions. In hypoxia, the effect of chloroquine was, however, significantly reduced (Fig. 2B). Similar effects on TLR9 mRNA expression by chloroquine were also detected in the D54MG and U373MG brain cancer cell lines (Fig. 2C and D). Furthermore, a similar trend in TLR9 mRNA expression was also detected in the Caco-2 and AGS human colorectal and gastric adenocarcinoma cell lines, respectively (Fig. 2E). At the protein level, however, chloroquine decreased MDA-MB-231 TLR9 protein expression, in normoxia and hypoxia (Fig. 2F). Similar effects on TLR9 protein were also detected in the control siRNA and TLR9 siRNA cells (Fig. 3). Taken together, these studies suggest that chloroquine has opposing effects on TLR9 mRNA and protein expression.

### Effects of chloroquine on MMP-2, MMP-9 and MMP-13 mRNA expression and proteolytic activity of TNBC cells with high and low TLR9 expression

**Figure 4:**
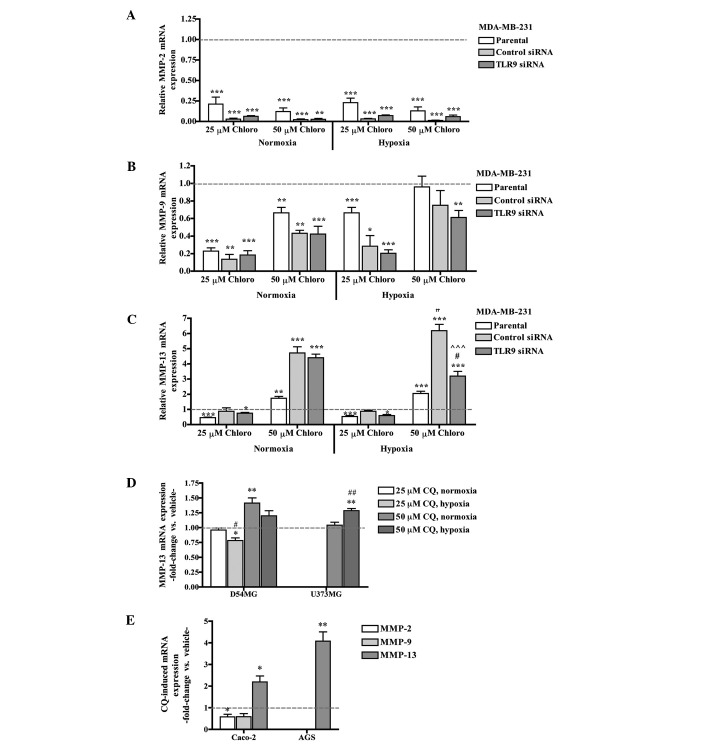
Expression of (A) MMP-2, (B) MMP-9 and (C) MMP-13 mRNA in parental MDA-MB-231 cells, control siRNA or TLR9 siRNA cells in normoxia and hypoxia, as measured with qPCR. The bars represent chloroquine-induced changes in mRNA expression, relative to vehicle-treatment (dotted line) in normoxia and hypoxia; mean ± SEM, n=3–6. ^*^P<0.05, ^**^P<0.01 and ^***^P<0.001 vs. the corresponding vehicle; ^#^P<0.05 vs. corresponding chloroquine in normoxia; ^^^^^P<0.001 vs. corresponding control siRNA. (D) D54MG and U373MG cells were cultured in normoxia and hypoxia in the presence of vehicle or 25 or 50 μM chloroquine for 24 h, and MMP-13 mRNA expression was measured with qPCR. Data is expressed as fold-change in MMP-13 mRNA expression vs. corresponding vehicle (represented by the dotted line). Mean ± SEM, n=6. ^*^P<0.05 vs. vehicle in normoxia, ^#^P<0.05 vs. vehicle in hypoxia, ^**^P<0.01 vs. corresponding vehicle in normoxia and ^##^P<0.01 vs. corresponding chloroquine-treatment in normoxia. (E) Caco-2 and AGS cells were cultured with 50 μM chloroquine in normoxia. Mean ± SEM, n=4. ^*^P<0.05, ^**^P<0.01 vs. corresponding vehicle. CQ, chloroquine; qPCR, quantitative PCR; MMP, matrix metalloproteinase; TLR9, toll-like receptor-9.

TLR9 ligand-induced invasion has been shown to be associated with the activation of MMP-13 (6–8). Since chloroquine inhibits TLR9-ligand-induced invasion in normoxia *in vitro*, the effects of chloroquine on MMP-2, MMP-9 and MMP-13 mRNA expression, as well as the proteolytic activity of TNBC cells with high and low TLR9 expression were investigated. Chloroquine had similar, suppressive effects on MMP-2 mRNA expression in normoxia and hypoxia in all the studied cells (Fig. 4A). The effects on MMP-9 mRNA expression were more dose- and oxygen-status dependent. While 25 μM chloroquine suppressed MMP-9 mRNA expression in normoxia and hypoxia, the 50-μM dose was less suppressive in normoxia and did not suppress MMP-9 mRNA expression in parental MDA-MB-231 cells under hypoxia. Similar effects were observed in the control and TLR9 siRNA MDA-MB-231 cells, with the exception that, in the TLR9 siRNA cells compared with vehicle-treatment, 50 μM chloroquine induced significant suppression of MMP-9 mRNA expression in normoxia and hypoxia (Fig. 4B). Chloroquine also had a dual, dose-dependent effect on MMP-13 mRNA expression. In normoxia and hypoxia, the 25-μM dose induced no change or slightly suppressed MMP-13 mRNA expression in all the studied cells. The higher chloroquine concentration (50 μM), however, induced a significant increase of MMP-13 mRNA expression in normoxia in all the cells. This induction of MMP-13 mRNA was further significantly enhanced by hypoxia in the control siRNA cells, but decreased in the TLR9 siRNA cells (Fig. 4C). Similar dose- and oxygen status-dependent effects on MMP-13 mRNA expression were also detected in the human D54MG and U373MG glioblastoma cell lines. The smaller dose had no or only a slightly suppressive effect on MMP-13 mRNA expression, while the higher dose induced MMP-13 mRNA in an oxygen level-dependent fashion (Fig. 4D). The Caco-2 and AGS cells were studied only in normoxia. In the Caco-2 cells, 50 μM chloroquine had no effect on MMP-9 mRNA, but suppressed MMP-2 mRNA and significantly induced MMP-13 mRNA expression. Similarly, 50 μM chloroquine also induced MMP-13 mRNA expression in the AGS cells (Fig. 4E). Taken together, these studies suggest that chloroquine has cell-, dose- and hypoxia-dependent effects on MMP-2, MMP-9 and MMP-13 mRNA expression. Most notably, higher doses of chloroquine appear to induce more MMP-13 mRNA expression, suppress less MMP-9 mRNA expression and, in hypoxia, these effect appear to be TLR9-dependent.

### Effects of chloroquine on MMP at the functional protein level

**Figure 5:**
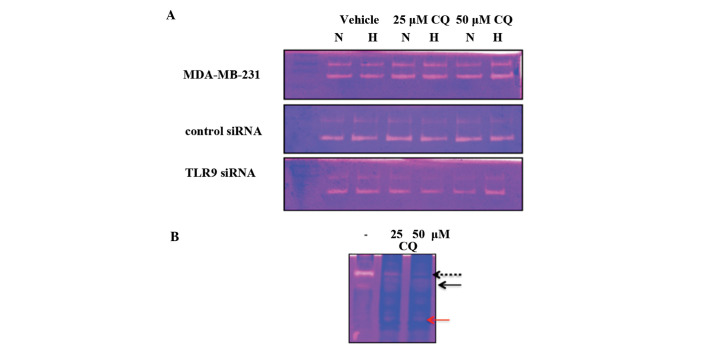
(A) Zymograms of supernatants from vehicle, 25 or 50 μM chloroquine-treated parental MDA-MB-231 cells or control siRNA or TLR9 siRNA cells after 24 h of treatment in normoxia (N) or hypoxia (H). (B) Zymogram of supernatants from vehicle, 25 or 50 μM chloroquine-treated TLR9 siRNA MDA-MB-231 cells in normoxia. The arrows point to the ~92 kDa pro-MMP-9 (dotted arrow) and the ~72 kDa pro-MMP-2 (black arrow). The ~40 kDa band represents MMP-13 (red arrow). MMP, matrix metalloproteinase; TLR9, toll-like receptor-9; CQ, chloroquine.

To investigate whether chloroquine’s effects on MMP mRNAs are translated to the functional protein level, zymograms were performed using the cell supernatants following the various treatments. Subsequent to 24 h of treatment, the pro-MMP-9 and pro-MMP-2 proteolytic bands were clearly visible (13), but no clear differences were detected in proteolytic activities between the various treatments of the studied cells (Fig. 5A). However, after 48 h, while MMP-2 and MMP-9 activities were suppressed by chloroquine, MMP-13 proteolytic activity began to emerge in the same specimens (Fig. 5B). Data is shown only for TLR9 siRNA cells in normoxia, although similar results were detected for all studied cells in normoxia and hypoxia.

### Anti-tumor efficacy of chloroquine in an orthotopic mouse model

**Figure 6:**
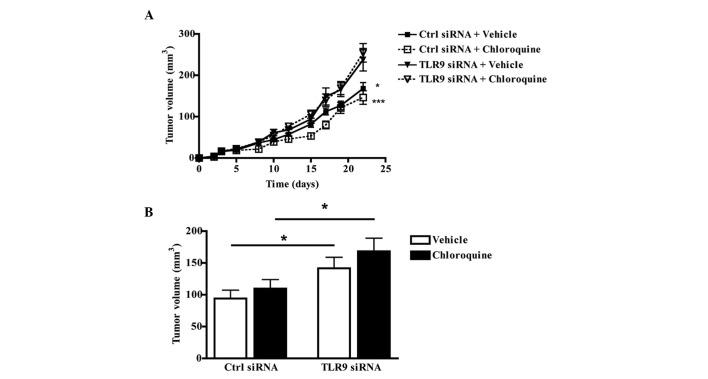
(A) Growth of orthotopic tumors formed by control siRNA or TLR9 siRNA MDA-MB-231 cells. Mean ± SEM, n=20. ^*^P<0.05 and ^***^P<0.01 TLR9 siRNA tumors vs. the corresponding value in the control siRNA tumors. (B) Tumor volumes at sacrifice, mean ± SEM, n=18–20. ^*^P<0.05, vehicle-treated TLR9 siRNA tumors vs. vehicle-treated control siRNA tumors, and chloroquine treated TLR9 siRNA tumors vs. chloroquine-treated control siRNA tumors. TLR9, toll-like receptor-9.

The anti-tumor efficacy of chloroquine was studied in an orthotopic mouse model, using control siRNA and TLR9 siRNA MDA-MB-231 cells. Subsequent to tumor cell inoculation and the establishment of tumors seven days later, the mice were treated daily with i.p. chloroquine (80 mg/kg). As expected, the TLR9 siRNA cells formed significantly larger tumors than the control siRNA cells during the experiment. Chloroquine treatment did not inhibit tumor growth in either the control siRNA or TLR9 siRNA groups (Fig. 6A and B). Taken together, despite the favorable antitumor and anti-invasive effects that chloroquine exhibits against the tested breast cancer cells *in vitro*, the results suggest that chloroquine does not prevent the growth of these cells at the orthotopic site *in vivo*.

## Discussion

In the current era of escalating cancer care costs, there is emerging interest in identifying new uses for old drugs (14,15). For example, chloroquine has demonstrated promising effects as an anti-cancer agent, particularly in breast cancers (16–19). Chloroquine has been shown to inhibit breast cancer growth *in vitro,* and low doses of chloroquine have induced resistance to mammary carcinogenesis in a rat model of chemically-induced breast cancers (20,21). Since our previous *in vitro* data suggested that chloroquine inhibits the invasive capacity of TNBC cells with the highly aggressive low TLR9 expression phenotype (7,8), the present study aimed to investigate the anti-tumor effects of this widely used, anti-malarial and rheumatology drug in a mouse model that mimics the aggressive human disease *in vivo*. According to our preliminary data, such patients with low TLR9-TNBC and poor prognoses may represent up to 10% of all breast cancer patients (9).

The present results demonstrated that, despite the promising hypoxia-associated growth inhibitory effects *in vitro*, chloroquine does not inhibit the local growth of tumors formed by the same cells *in vivo*. The reason for the discrepancy between the *in vitro* and *in vivo* findings is currently unclear and requires further characterization. Local tumor growth is the sum of cell proliferation and local invasion. Thus the lack of inhibition of tumor growth may, at least partially, be explained by the pro-invasive effects of chloroquine, such as increased MMP-13 activity, which at the protein level manifests later than the anti-invasive and growth-inhibitory effects and which may be more pronounced in hypoxic conditions. The present results are the opposite of those published by Jiang *et al*(22), who observed that chloroquine inhibits the growth of subcutaneous (s.c.) 4T1 breast tumors and lung metastases *in vivo*. Chloroquine, alone or in combination with the mTOR inhibitor RAD001, has also been shown to inhibit the *in vivo* growth of orthotopic MCF-7 tumors (21). The differences in the results may be explained by the different cell lines used and the drug dosage; it is possible that, for example, the MMP-13-activating effects of chloroquine manifest only with the higher chloroquine doses, similar to those used in our studies. A part of the differences in chloroquine responses may also be explained by the p53 status of the cell lines used. Chloroquine is known to induce cell cycle arrest through the activation of the p53 tumor suppressor, which is mutated in MDA-MB-231 cells (20). The present results, which showed that chloroquine inhibits the hypoxia-induced increased viability of these cells, suggest that in hypoxia, chloroquine may induce other pathways of cell death or growth arrest, independent of p53. This is supported by the fact that the 4T1 cells have been shown to be p53 null (23).

TLR9 is a cellular DNA receptor which, based on our observations, appears to regulate cancer cell invasion in the absence of exogenously added DNA ligands. Chloroquine has been shown to inhibit TLR9 ligand-induced inflammatory reactions in cells and this effect has been attributed to the inhibition of endosomal acidification and more recently, to direct binding of chloroquine to nucleic acids, thus masking their TLR9-binding epitopes (24). Notably, the present study revealed that chloroquine treatment upregulates TLR9 mRNA expression in cancer cells. This effect on mRNA was slightly reduced in hypoxia, but did not translate into increased TLR9 protein levels, even in oxygen replete conditions. By contrast, chloroquine treatment actually resulted in decreased TLR9 protein expression. The present results agree with those of Zhu *et al*(25) who demonstrated that chloroquine inhibits TLR9 expression in dendritic cells. The reason for these findings is unclear, but it may be that a low pH is required for the proper folding of the TLR9 protein or the involvement of specific microRNAs that would inhibit TLR9 expression. By blocking the acidification of the endosomal organelles where TLR9 resides, chloroquine may also actually hasten the degradation of the TLR9 protein. Although Kuznik *et al*(24) demonstrated that chloroquine does not increase the pH of endosomes, the concentration of chloroquine these authors used was significantly smaller compared with the present experiments (4 vs. 25–50 μM). These issues require further biochemical characterization at the cellular level. Another possible explanation for why chloroquine does not prevent tumor growth in this model is that reducing TLR9 expression may promote the highly aggressive low TLR9 expression phenotype of the TNBC cells (9), thus allowing the activation of the presently unknown pathway of aggressive growth and invasion.

In conclusion, despite the promising TLR9 status-independent growth inhibitory and anti-invasive *in vitro* effects against TNBC cells in normoxia and hypoxia, chloroquine does not inhibit the growth of orthotopic TNBC tumors *in vivo*. Furthermore, by promoting MMP-13 activation and suppressing TLR9 expression under such conditions, chloroquine may be a particularly poor choice for tumors that are hypoxic. Chloroquine may, however, have growth inhibitory and anti-metastatic effects against other types of breast or other cancers.
